# Spontaneous Retroperitoneal Haemorrhage From Bilateral Renal Angiomyolipomas: A Case Report on a First Presentation of Tuberous Sclerosis Complex

**DOI:** 10.7759/cureus.95129

**Published:** 2025-10-22

**Authors:** Christian J Kirk, Timothy Burns, Flavio Ordones, Wikus Vermeulen

**Affiliations:** 1 Urology, Bay of Plenty District Health Board, Tauranga, NZL

**Keywords:** case report, embolisation, pseudoaneurysm, renal angiomyolipoma, retroperitoneal haemorrhage, tuberous sclerosis complex

## Abstract

Renal angiomyolipomas (AMLs) are benign mesenchymal tumours that may present with life-threatening haemorrhages when associated with underlying genetic conditions. AMLs are comprised of mature but disorganised vasculature, smooth muscle and fatty adipose tissue. Whilst the majority of intrarenal AMLs are sporadic and found incidentally as solitary lesions, there is a significant association with tuberous sclerosis complex (TSC).

TSC results in the disruption of the mTOR pathway, leading to unregulated cell proliferation and subsequent benign tumour growth. Consequently, AMLs present in TSC are typically large, multiple and complex in nature.

In this case, we report a 22-year-old woman with no significant past medical history, who presented with right‑sided abdominal pain and was initially investigated for cholecystitis. Imaging revealed bilateral renal AMLs, a right renal artery pseudoaneurysm and acute retroperitoneal haemorrhage. The patient was stabilised with a transfusion and proceeded to undergo emergency super‑selective embolisation. Physical examination and radiological clues raised further suspicion of TSC.

Follow-up interval imaging revealed a 9 mm intra-tumoral aneurysm in the contralateral kidney, which was prophylactically embolised, and neuroimaging revealed subependymal nodules. Subsequent treatment is anticipated to involve pharmaceutical mTOR inhibition, definitive neurological assessment and follow-up at the regional Genetics Centre.

In patients with complex AMLs and clues of genetic disease, evaluation for TSC should be prompt. The presence of secondary features should raise suspicion and initiate assessment of underlying genetic conditions in young patients. Evaluation may then unmask serious complications, such as haemorrhage, and early recognition enables targeted intervention, surveillance and holistic patient counselling.

## Introduction

Angiomyolipomas (AMLs) are hamartomatous lesions. They originate from mesenchymal tissue and contain smooth muscle, adipose tissue and fragile, dysmorphic vessels that predispose to aneurysmal formation and rupture [1].

Solitary sporadic renal AMLs are found incidentally in up to 3% of the general population, predominantly in middle‑aged women [1]. In contrast, up to 80% of patients with tuberous sclerosis complex (TSC) develop multiple, bilateral renal AMLs that tend to be larger, more numerous and subsequently more prone to spontaneous haemorrhage [2]. The risk of haemorrhage of renal AMLs in TSC patients has been reported between 25% and 50% [3].

TSC is an autosomal dominant neurocutaneous disorder caused by mutations in the TSC1 or TSC2 gene, with a birth prevalence of roughly 1:20,000 [4]. The TSC1 and TSC2 genes are intrinsically related to AMLs, as they are responsible for the production of proteins, hamartin and tuberin, respectively. In TSC, the mTOR pathway becomes unregulated, and the mutations manifest as uncontrolled proliferation in disorganised mature cells, which are endemic to the local site or organ [5,6].

There is a significant neurological component, with 70% of patients being epileptic. Its relationship with AMLs are also exhibited extra-renally as hepatic or splenic lesions; infrequently, pulmonary lesions in the form of lymphangioleimyomatoses (proliferation of smooth muscle cells) are also found [7].

This report highlights a rare but serious presentation of bilateral intrarenal AMLs with spontaneous bleeding in a young woman and underscores the importance of recognising secondary features suggestive of TSC.

## Case presentation

**Figure 1:**
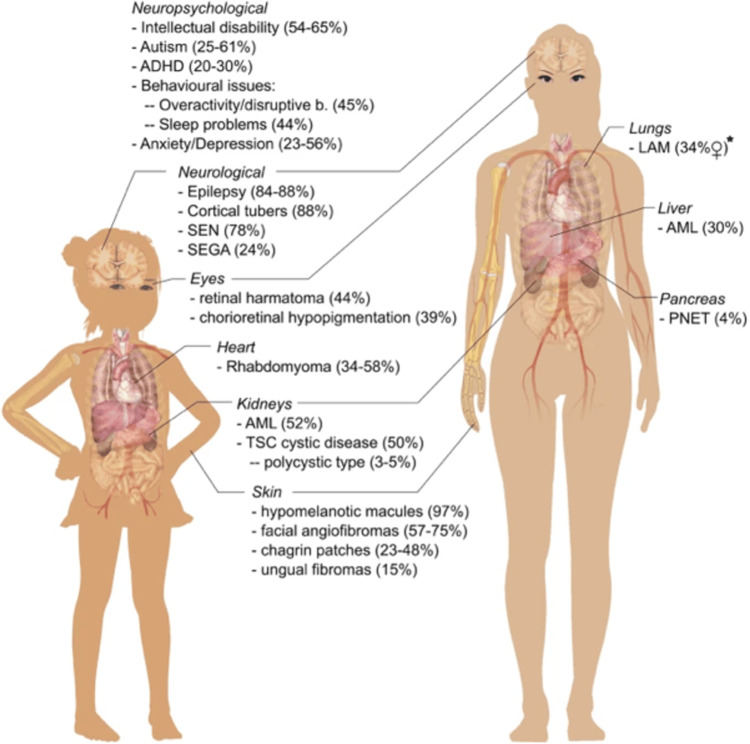
Visual representation of the clinical manifestations and systemic sequelae of tuberous sclerosis complex Source: [8]

The systemic sequelae listed earlier are complex and visually summarised in Figure 1. 

Patient information and clinical findings

This case discusses a 22-year-old female who presented with a 24-hour history of nausea, vomiting and right-sided abdominal pain, with no preceding history of trauma. Upon questioning, the patient had no relevant past medical history, took no regular medications and had no significant or known family history. 

**Figure 2:**
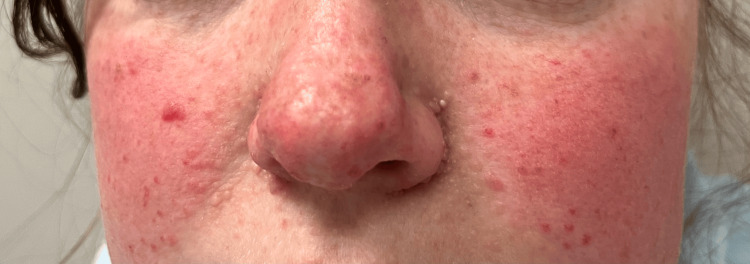
Facial angiofibromas: cutaneous manifestation of tuberous sclerosis complex The figure highlights the presence of facial angiofibromas. These can be found in the major diagnostic criteria described by the Tuberous Sclerosis Complex Consensus Guidelines [9].

Upon review of her observations at the time of her admission, all were within normal limits. The patient was then examined, which revealed right upper-quadrant and right flank tenderness, but no palpable masses. Multiple facial angio-fibromas were noted as part of her examination; these are pictured in Figure 2. 

Table 1 outlines the chronology of events of this patient's admission.

**Table 1:** Timeline of events AML: angiomyolipoma; CTKUB: computed tomography of kidneys, ureters and bladder; TSC: tuberous sclerosis complex

Time Period	Event
Day 1	Presentation to the Emergency Department and admission
	US Abdomen – echogenic fat identified across both right and left renal regions
Day 2	11:00 – MRI shows bilateral renal AMLs, haemorrhage and hepatic AML
	16:00 – Triple-phase CTKUB shows a 9 mm right renal artery pseudoaneurysm and confirms right-sided retroperitoneal haemorrhage
	18.00 – Emergency super-selective embolisation & 1 RBC unit
Day 3	Bed rest and monitoring
Day 4	Discharged home
Week 6	Follow-up imaging included CTCAP, triple-phase CTKUB, and CT brain. The imaging was used to assess pseudo-aneurysm resolution, monitor AML stability and evaluate intracranial TSC-associated lesions, following standard post-embolisation and multi-system surveillance protocols
	Follow-up discussion in clinic with results and referral pathways
Month 3	Genetic counselling and specialist follow-ups for TSC

Diagnostic assessment

Table 2 outlines the patient's biochemistry results from arrival and across admission. Upon arrival, bloods showed a mild normocytic anaemia, which progressed rapidly from 109 to 79g/L across the course of 24 hours on June 6, 2025. White blood cell (WBC) remained within normal ranges throughout admission, in addition to renal function monitoring.

**Table 2:** Serum biochemistry results Result interpretation summarised in the following text. MCV: mean corpuscular volume; eGFR: estimated glomerular filtration rate

	5^th^ June 2025	6^th^ June 2025	6^th^ June 2025	7^th^ June 2025	8^th^ June 2025	12^th^ August 2025	Units	Ref Ranges
		17:17	18:56					
Hb	109	79	80	80	91	115	g/L	115-155
MCV	83	84	82	82	84	83	fL	80-99
WBC	10.9	7.8	8.9	9.2	8.1	4.1	x10E9/L	4.0-11.0
Platelet Count	409	195	223	191	273	326	x10E9/L	150-400
Creatinine	52	-	-	56	53	55	umol/L	45-90
eGFR	>90	-	-	>90	>90	>90	mL/min	>60

The sudden drop in haemoglobin noted across the course of June 6 returned subsequent to the completed MRI images, which prompted further evaluation for haemorrhage. The return of haemoglobin to acceptable levels, as seen in Table 2, followed red blood cell transfusions and definitive embolisation.

**Figure 3:**
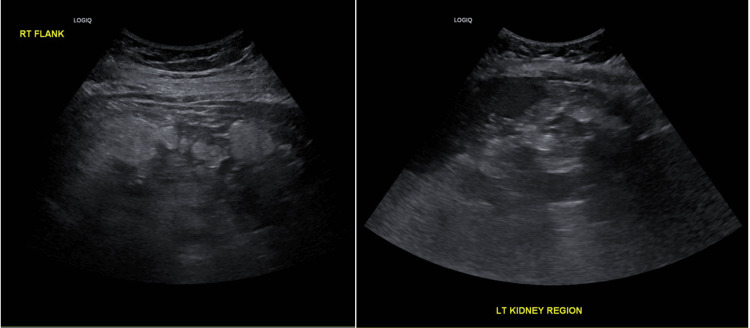
US abdomen: views of right and left kidney regions Ultrasound (US) abdomen images show large echogenic fat deposits across the kidney regions.

An ultrasound (US) scan of the abdomen was completed during emergency assessment to exclude cholelithiasis on June 5. Figure 3 shows this, with both right and left renal images. Echogenic fat was identified superficial to the expected positions of both kidneys. The gallbladder was determined to be thin-walled, with no evidence of cholelithiasis. Due to the limitations of the US images, further imaging was recommended for optimal visualisation.

**Figure 4:**
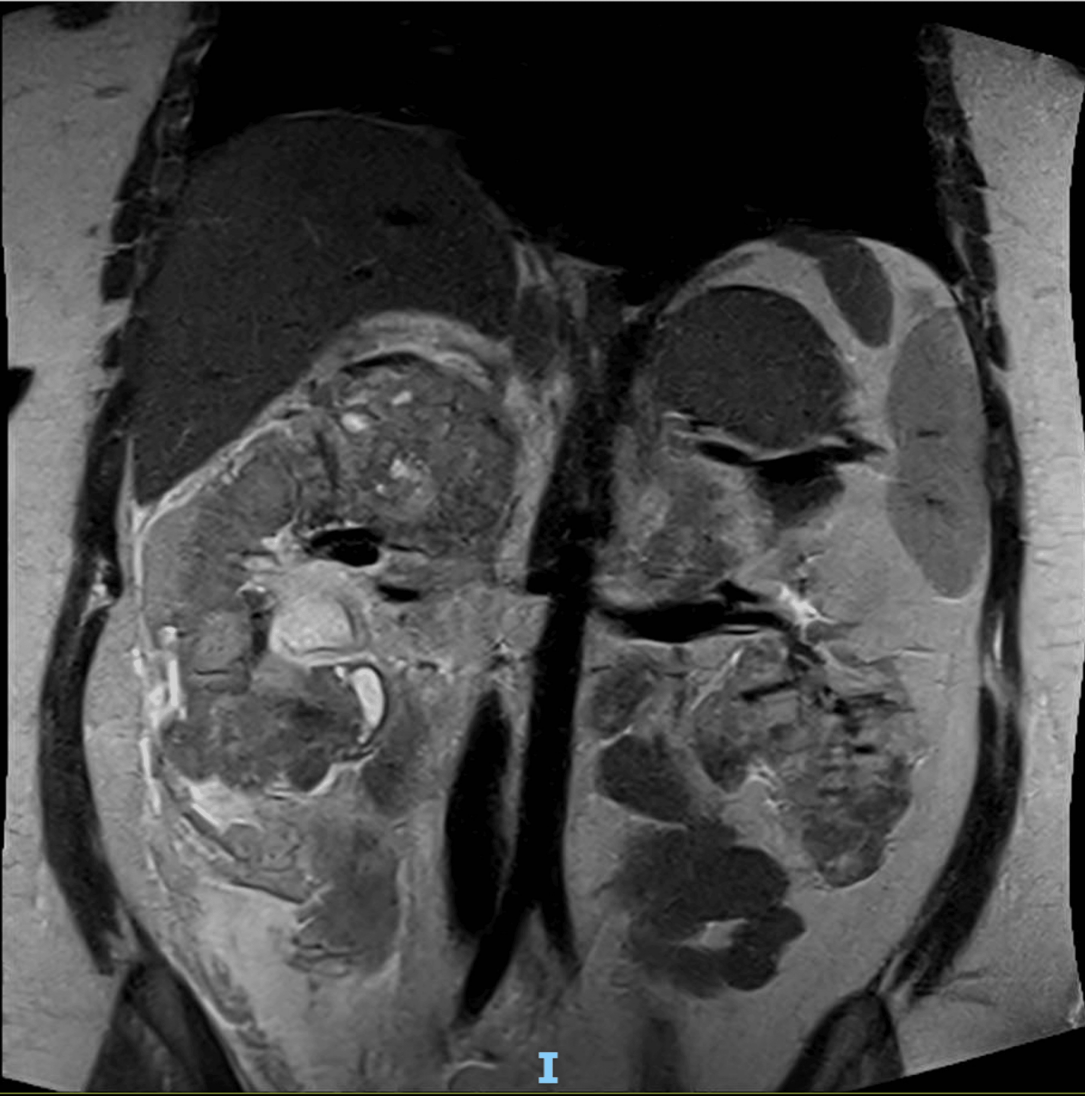
MRI abdomen T2-weighted: coronal thin slice The MRI shows grossly distorted kidneys and innumerable bilateral intrarenal angiomyolipomas.

**Figure 5:**
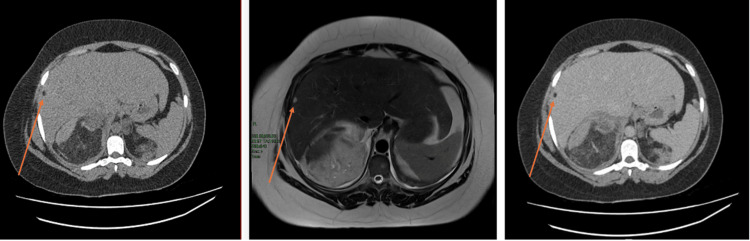
Axial views of hepatic AML in CT kidney triple phase (non-contrast phase), MRI abdomen T1-weighted and CT kidney triple phase (contrast-enhanced portal venous phase) The figure shows axial views with arrows highlighting the reported 8 mm focal hepatic angiomyolipoma within segment VIII of the liver. This is seen first across the non-contrast CT image (image on the left), with a corresponding lesion on the second T1-weighted MRI image (central). The final image is contrast-enhanced, and the lesion is again highlighted (image on the right). AML: angiomyolipoma

The ultrasound image was followed up with magnetic resonance imaging (MRI) of the abdomen. Marked abnormalities of both kidneys are clearly imaged in Figure 4 and were reported as likely extensive bilateral angiomyolipomas. The small volume of free fluid was identified as a probable haemorrhage, and triphasic renal computed tomography (CT) was recommended for the next stage of assessment. A focal, concurrent, 8 mm fat-signal lesion was also pictured within segment VIII of the liver, possibly representing a hepatic AML. This is shown in the CT (both enhanced and non-contrast enhanced) and T1-weighted MRI images in Figure 5.

**Figure 6:**
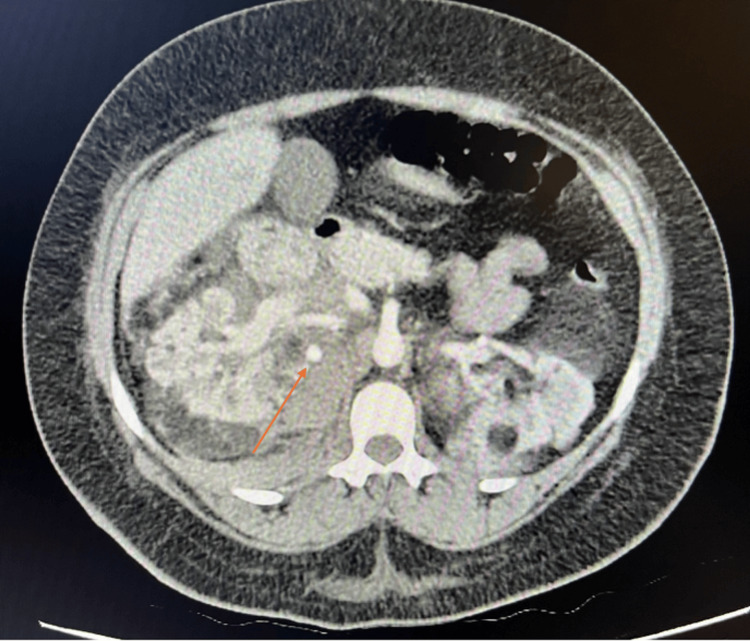
Index CT kidney triple phase: arterial phase with axial views of the abdomen The arrow highlights the right renal artery pseudoaneurysm as a hyperdense circular abnormality.

**Figure 7:**
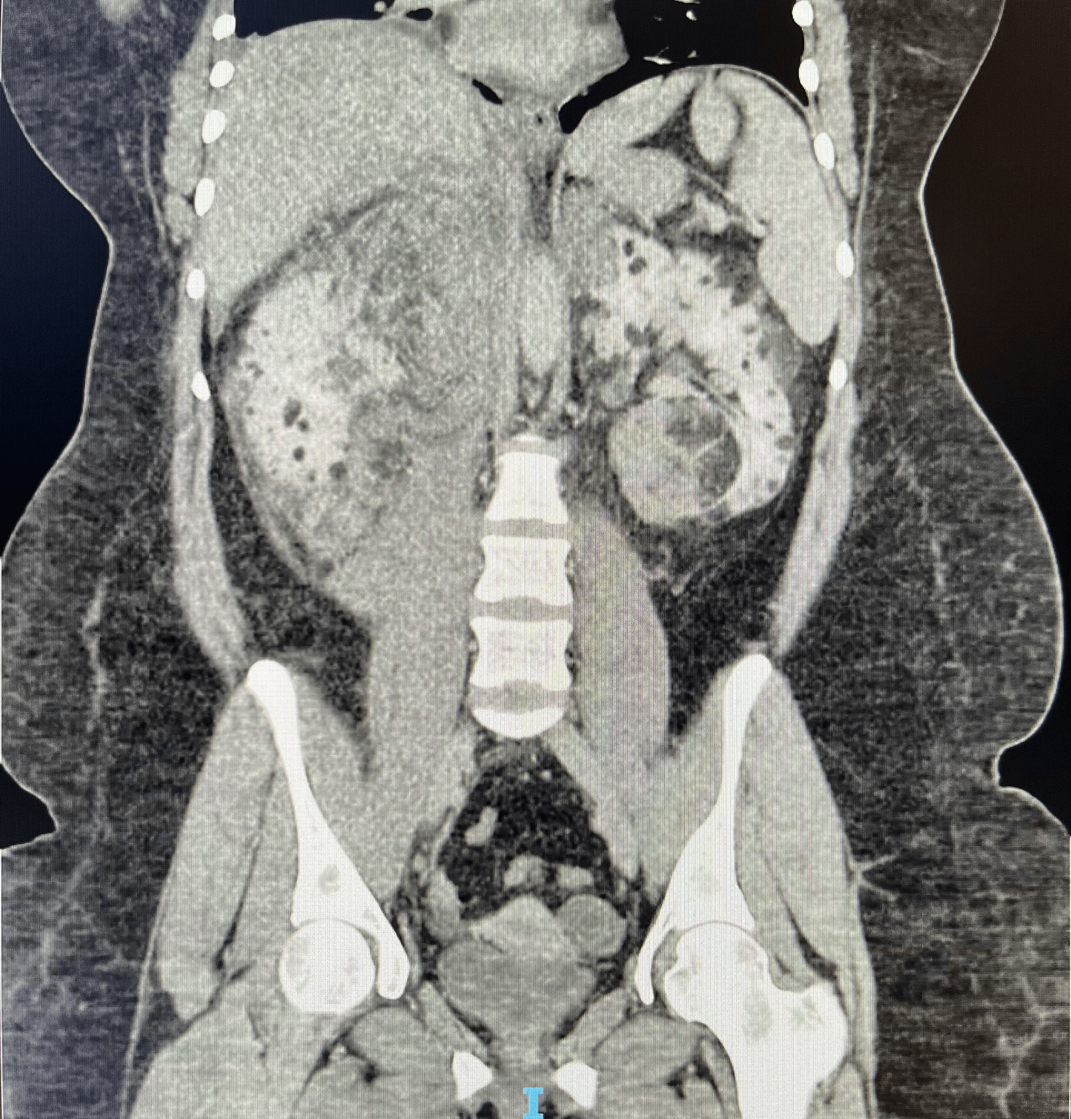
Index CT kidney triple phase: arterial phase with coronal views of the abdomen The figure highlights the extent of intrarenal AMLs. There are innumerable bilateral renal angiomyolipomas with multiple intra-tumoral haemorrhages, as demonstrated in the inferior pole of the left kidney. AML: angiomyolipoma

**Figure 8:**
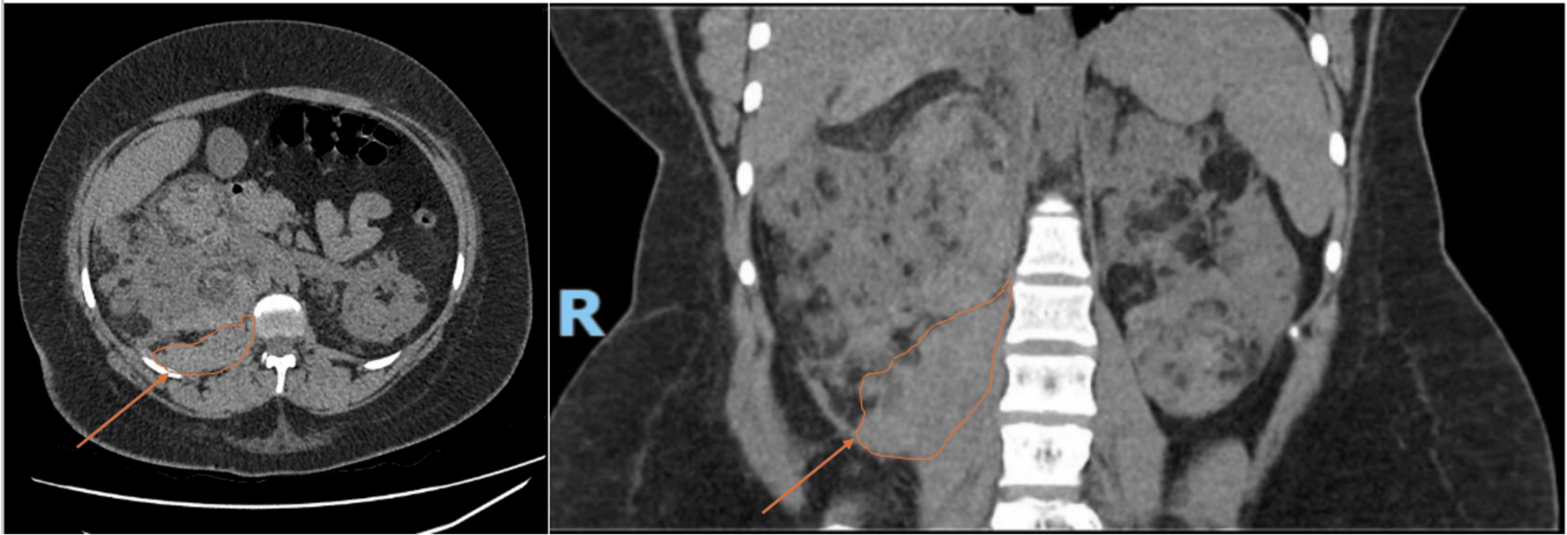
CT kidney triple phase: non-contrast enhanced phase with axial and coronal views of the retroperitoneal haemorrhage The figure outlines the extent of the right-sided retroperitoneal haemorrhage, which lies inferior to the right kidney and lateral to the iliopsoas muscle. The grossly abnormal renal architecture can also be appreciated with innumerable intrarenal angiomyolipomas bilaterally.

Figure 6 and Figure 7 represent axial and coronal views from the triphasic CT scan. Numerous AMLs with adjacent hyperdense haemorrhage are visualised, alongside a 9 mm arterial blush consistent with a pseudoaneurysm as seen in the axial plane. Figure 8 shows the extent of the retroperitoneal haemorrhage across axial and coronal views of the non-contrast CT images, extending from the right ruptured pseudoaneurysm inferiorly below the right kidney.

Therapeutic intervention

Emergency trans‑arterial embolisation was performed via retrograde access through the right common femoral artery. A microcatheter was advanced to the lower branch vessel of the right renal artery feeding the pseudoaneurysm. Embolisation via a combination of 0.18 micro‑coils and 0.35 regular coils achieved complete haemostasis and control of the pseudoaneurysm. One unit of packed red blood cells was transfused post-procedure.

Follow-up and outcomes

The patient remained haemodynamically stable post‑embolisation with regular observations. She was discharged on Day 4 with analgesia and instructions for limited activity.

**Figure 9:**
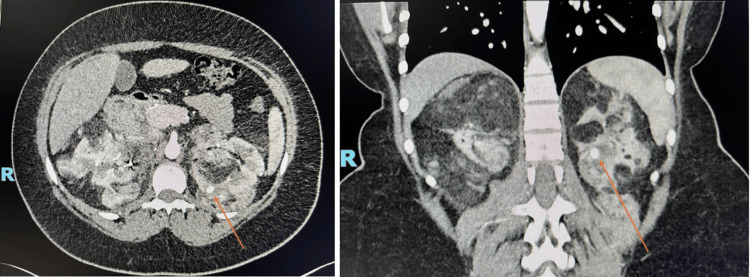
Follow-up CT imaging: arterial phase with coronal and axial views of the kidneys The arrows highlight a left 9 mm intra-tumoral aneurysm for prophylactic embolisation due to high risk of rupture. This is the hyperdense circular abnormality.

A follow-up set of CT imaging was completed at six weeks to assess the resolution of the renal pseudo-aneurysm, the stability of the AML burden and to guide further speciality input. Both axial and coronal views are presented in Figure 9. The imaging confirmed resolution of the previously haemorrhaging right-sided pseudo-aneurysm. However, the finding of a further 9 mm intra-lesional aneurysm within the left kidney prompted referral for a second embolisation as a prophylactic measure. An orange arrow highlights this in Figure 9. 

**Figure 10:**
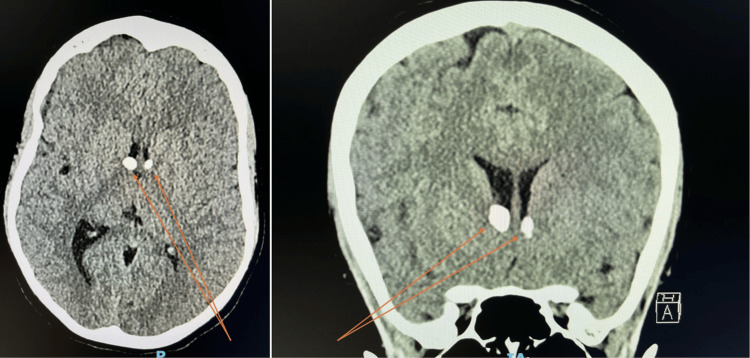
Follow-up CT imaging: axial and coronal views of the brain Arrows highlight calcified subependymal nodules within the lateral ventricles of the brain. These are recognised intracranial sequelae of TSC. Follow-up MRI imaging was requested for superior characterisation ahead of specialist neurological input.

Figure 10 highlights both axial and coronal views of the CT head. Multiple sub-ependymal nodules within the lateral ventricles of the brain are noted; these are recognised features in TSC.

Imaging of the chest identified cardiothoracic lesions, with a 16 mm fatty lesion within the septal wall of the left ventricle of the heart. Small lung cysts were identified; however, based on radiological characteristics (size, distribution, cyst wall appearance and absence of chylous effusions), these were considered unlikely to represent lymphangioleiomyomatosis, a known pulmonary manifestation in TSC.

Elsewhere, the hepatic AML pictured in Figure 5 on initial imaging was again identified, in addition to sclerotic bony foci found within the pelvis; these are not pictured in the above figures.

The results of the latest images and the range of lesions identified were discussed in an outpatient clinic setting with the patient and their relatives. Further speciality referrals and the need for discussion within a multidisciplinary meeting were highlighted at the time. On further questioning, there was no personal history of seizures and no known family history of the condition, although a family history of a relative passing at a young age from seizures was reported. In addition to individual specialist referrals, such as for consideration for mTOR inhibitor therapy, a referral was also made to the Genetics Department to examine familial connections in more detail.

## Discussion

Counselling young women with the diagnosis is particularly important, as pregnancy is responsible for a recognised boost in AML growth and subsequently haemorrhage. Two main factors drive this. The first is structural: during pregnancy, oestrogen and progesterone circulate within the blood in high volume. AMLs express oestrogen and progesterone receptors and subsequently respond with accelerated cell growth and smooth muscle proliferation. This results in increased haemorrhage risk, as AMLs are hamartomas and so lack the typical elastic lamina that would be found in an organised tissue structure [10]. This leaves the rapidly growing tumours fragile and prone to aneurysmal formation.

The second component is related to haemodynamics. During pregnancy, renal blood flow increases between 50% and 80%, as the maternal body compensates for the foetus. This is a consequence of systemic vasodilation to improve renal perfusion, increased cardiac output and hormonal response to oestrogen and progesterone [11]. This increases intravascular stress on the abnormal tissue structure and results in a higher risk of spontaneous AML rupture [12].

As a consequence of these factors, pre-pregnancy counselling and screening in women with TSC should be completed. This typically entails annual renal function and blood pressure monitoring, baseline renal MRIs to assess AML size and growth rates, and pulmonary assessment via chest high-resolution CT and pulmonary function testing for lymphangioleiomyomatosis (LAM). In women of child-bearing age, high-risk AMLs should be stabilised prior to conception to reduce the risk of rupture [13,14].

In TSC, AMLs are often larger, bilateral and multifocal, further increasing haemorrhage potential [2]. Lesions >4 cm or with intra-lesional aneurysms carry the highest bleeding risk [15]. The threshold of 4 cm, representing a higher bleeding risk and, as a result, an indication for intervention, originates from a combination of observational and prospective studies. In a report in 1986, patients were found more likely to experience symptoms and spontaneous haemorrhage with larger AMLs, specifically >4 cm. Larger AMLs understandably carried a greater bleeding risk [16]. Since then, alternative indicators for prompting intervention have been suggested, such as intralesional aneurysm size >5 mm [17].

Spontaneous retroperitoneal haemorrhage is a urological emergency. Super‑selective arterial embolisation is the preferred first‑line surgical intervention because it is minimally invasive, kidney‑sparing and highly effective in achieving haemostasis [18]. Alternative surgical options, such as partial or total nephrectomies, may be required in cases refractory to embolisation; however, they should ideally be avoided due to unnecessary loss of existing healthy tissue and the risk of compromising future renal function [9].

There is a recognised role for the conservative management of AMLs. Active surveillance is the mainstay, given that the vast majority of lesions remain small and asymptomatic [19].

For patients not suited to surgery or as an alternative intervention for complex AMLs, medical therapy such as mTOR inhibitors, e.g., Everolimus, could be considered. In patients with TSC, mTOR activity is unregulated, promoting cell proliferation and angiogenesis. Targeting this pathway results in reduced tumour growth and reduced haemorrhage risk. The 2012 International Tuberous Sclerosis Complex Consensus guidelines now recommend mTOR inhibitors as the first-line treatment; current guidelines recommend considering medical therapy for asymptomatic AMLs ≥3 cm in diameter [9,20].

Beyond acute management, recognition of TSC is critical. The dermatological stigmata (e.g. facial angiofibromas) and radiological findings of extra‑renal AMLs or associated lesions (hepatic, splenic, pulmonary), as for in this patient, raised diagnostic suspicion. This warranted an investigation into multi-speciality input and lifelong surveillance for renal, neurological and extra-renal sequelae.

## Conclusions

Angiomyolipomas are benign tumours that have the propensity for aneurysmal formation and rupture. The risk of rupture of intrarenal AMLs increases where they are large, multiple, have intra-lesional aneurysmal components or in the context of pregnancy.

This case emphasises the importance of maintaining a high index of suspicion for underlying genetic disorders, in addition to prompt recognition of the requirement for emergency treatment, such as in the presence of acute haemorrhage as described in our case. The spectrum of clinical manifestations that complex genetic conditions may present with are also highlighted. TSC patients typically have a significant neurological history, with the predominant feature being epilepsy. This case highlights recognition of outlier patients who present with no significant clues in their past medical history, but require life-preserving treatment. Skilled and empathetic communication are essential for clinicians finding themselves in the position of needing to acutely recognise and treat an emergency, in addition to broaching a significant and life-altering diagnosis with their patient. This is particularly true for women of childbearing age requiring pre-pregnancy counselling or patients facing multiple and repetitive surgical interventions. Early identification of TSC following acute intervention facilitates appropriate long-term multi-speciality surveillance, genetic counselling and consideration of targeted mTOR inhibitor therapy alongside interventional radiology or surgery.
